# Remoteness‐attributable financial inequality in dental service utilization in Australian older adults: A Blinder‐Oaxaca decomposition

**DOI:** 10.1111/cdoe.13004

**Published:** 2024-08-18

**Authors:** Arash Ghanbarzadegan, Xiangqun Ju, Woosung Sohn, Lisa Jamieson

**Affiliations:** ^1^ Australian Research Centre for Population Oral Health (ARCPOH) Adelaide Dental School, The University of Adelaide Adelaide South Australia Australia; ^2^ Population Oral Health, Sydney Dental School, Faculty of Medicine and Health The University of Sydney Sydney New South Wales Australia

**Keywords:** aged, dental health services, healthcare disparities, residence characteristics, rural population

## Abstract

**Objective:**

Oral health is often overlooked in ageing health issues, despite its impact on overall health and quality of life. Older Australians, especially those in rural and remote areas, face difficulties accessing oral health services. The aim of the study was to investigate the factors that contribute to financial barriers to accessing dental services among the ageing population in Australia in relation to their residential location.

**Method:**

The study included a weighted sample of Australian adults aged 65 years and over from a population‐based survey called the National Study of Adult Oral Health (NSAOH) conducted in 2017–18. Descriptive analysis was conducted and generated cross‐tabulation tables to investigate the distributions of the outcome, exposure and covariates, including Sex, Education level (the highest level of education), Equivalised household income, Dental insurance, Concession card ownership, Difficulty paying a dental bill and last dental visit. Blinder‐Oaxaca decomposition counterfactual analysis was used to explore the potential impact of a person's residence on their financial difficulty accessing dental services.

**Results:**

The findings showed that 26.2% (95% CI: 24.3–29.3) of major city residents and 30.1% (95% CI: 26.9–33.3) of rural residents avoided or delayed dental visits due to cost. The decomposition analysis indicated that 53.8% of the disparities in the prevalence of avoided or delayed dental visits due to cost were explained by the selected variables, while 46.2% remained unexplained. The explanatory variable with the largest contribution was difficulty paying a $200 dental bill, accounting for 62.4% of the differences, followed by dental insurance, last dental visit and equivalised household income, which explained 42.1%, 20.8% and 14.9% of the differences, respectively.

**Conclusion:**

Regional/remote populations experience more financial barriers to accessing dental care than major city populations and the identified factors explain a significant proportion of these disparities. Based on the study findings, recommendations include expanding public dental service coverage, evaluating concession card mechanisms and advocating for regular dental visits to mitigate disparities in dental care access.

## 1 INTRODUCTION

The fast growth of the ageing population in developed countries has made this population's needs and quality of life essential concerns of healthcare professionals and policymakers. Ageing brings physiological and physical changes leading to numerous health problems.^1^, ^2^ Aligned with these concerns, the United Nations (UN) General Assembly announced 2021–2030 the UN Decade of Healthy Ageing, encouraging the global collaboration of governments to provide longer and healthier lives for the aged population.^3^ However, when it comes to ageing health issues, oral health is overlooked.^4^

Similar to other developed countries, Australia has a high median age, with a considerable share of its population aged 65 and over. In 2020, 4.2 million Australians were 65 years old, which accounted for 16% of the general Australian population.^5^ This number is anticipated to increase by 2066 when older Australians contribute 21%–23% of the total population.^5^

Rural Australians' mortality rate is 1.3 times higher than the rate for urban Australians.^6^ This could reflect the fact that rural and remote Australians are more exposed to risk factors associated with chronic diseases, such as excessive weight, smoking, and diabetes.^7^ Similarly, there is lower access to health services in rural areas.^8^ This higher disease burden, greater prevalence and lower access are more significant in rural residents.^7^, ^8^ This also extends to oral health services, which is more pronounced in older Australians. Older Australians more often experience oral diseases and difficulty accessing oral health services.^8^, ^9^

Younger individuals often choose to live in major cities, attracted by educational and job opportunities, as well as convenient access to social activities. Conversely, older individuals may also relocate to major cities, driven by the desire for improved access to services.^10^

According to the Australian Institute of Health and Welfare, approximately 4.1 million individuals aged 65 and over are currently living in Australia. Among this demographic, two‐thirds, equivalent to 66% or 2.7 million, reside in major cities, while the remaining 34% (1.4 million) are situated in rural/remote areas.^11^ In comparison to the overall Australian population, a greater percentage of older individuals resided in rural/remote areas, while a smaller percentage lived in major cities.^7^, ^11^

This uneven distribution underscores the imperative for reform in aged care. While there have been some broad government initiatives like Community Aged Care Packages, unfortunately, these services are restricted primarily to personal care, social support, transportation to appointments, home assistance, meal preparation and gardening.^12^ There are still gaps in accessing health services beyond the coverage provided by the general population's Medicare (Australian public insurance).^13^

Notably, when it comes to public dental services, government subsidies are comparatively limited compared to other health services, with no specific dental benefit package tailored for older adults.^14^, ^15^ Only adults, including the elderly, who have access to government concession cards may be eligible to receive public dental services, primarily located in major cities, but often entailing extended waiting times.^14^

Additionally, income inequality is a significant issue for the more than 6.7 million people in rural and remote Australia.^8^ Access to health care services, housing, and education in rural and remote Australia are vital concerns in these areas, increasing the risk of chronic diseases among the people of rural and remote Australia.^9^ Despite the worse health condition, the ageing population are more likely not to attend their medical and dental visits, treatments, tests and medications because of unaffordable cost.^8^

Given the mentioned health challenges faced by the ageing population, including the overlooked aspect of oral health and the compounded difficulties experienced by rural residents due to limited access to healthcare services and limited government subsidies, it is essential to investigate this issue further. With the growing ageing population in both global and Australian contexts, particularly in rural areas, this study aims to explore the effect of residential location on financial delays or avoidance of dental services among older Australians. Specifically, the research question is:

How does residential location affect the financial delays or avoidance of dental services among older Australians?

To address this research question and aim, the study has the following objectives:

1. To determine the prevalence of delayed or avoided dental visits due to cost among older Australians in different residential locations.
2. To identify and compare the sociodemographic and economic factors contributing to financial delays or avoidance of dental services among older Australians based on residential location.

This investigation will help to address the gap in understanding and improve oral health outcomes for this vulnerable demographic.

## 2 METHODS

The National Study of Adult Oral Health (NSAOH) was a population‐based cross‐sectional survey of Australian adults aged ≥15 years, which was conducted in 2017–18.^16^ In NSAOH, a representative sample of Australians was drawn through a three‐stage stratified sample design within each state/territory. A sampling frame was created (including all Australian in‐scope postcodes) in the first stage; 15 strata were defined from a postcode sampling frame in the second stage; and adults aged ≥15 years were selected randomly from each sample household to participate. Self‐reported information about oral health and related characteristics were collected using a computer‐assisted telephone interview (CATI) or online questionnaire. Key variables included oral health status, access to dental services, financial barriers, dental insurance, sociodemographic details, and health service utilization. The study examined a weighted sample of adults aged 65 and over from NSAOH, using a multi‐stage design that considered diverse probabilities in selecting postcodes, households and individuals. Weights were calculated to adjust for participation variations across postcodes, age groups and sex categories, ensuring representative estimates aligned with the Australian population's demographics.^16^

## 3 VARIABLES

### 3.1 Outcome: avoided or delayed visiting due to cost

Participants were asked whether they had avoided or delayed visiting a dental practitioner in the last 12 months due to cost. The response options were No (=0) or Yes (=1).

Exposure: Residential location.

This variable has been identified as a significant determinant of dental service availability, as the majority of public dental services and a higher concentration of dentists are found in major cities.^6^, ^17^ It was geographically classified as very remote/remote, outer regional, inner regional or major cities based on the Australian Bureau of Statistics—Greater Capital City Statistical Area (GCCSA) classification and derived from the postcodes used in NSAOH. Residentical location, then, was dichotomised into ‘Rural/remote’ (including remote/remote, outer regional, inner regional) and ‘Major city’.

### 3.2 Covariates

Models were adjusted for several covariates, including Sex, Education level (the highest level of education), Equivalised household income, Dental insurance, Concession card ownership, Difficulty paying a dental bill and last dental visit. Participants' gender was coded as Male (=0) or Female (=1). The selection of variables was primarily informed by the Aday and Andersen framework for health access^18^ and cross‐checked against both Australia's National Oral Health Plan and its Performance Monitoring Report.^19^, ^20^

Education level was derived from the question ‘What is the highest qualification or level of education you have completed?’ It was categorized into secondary school or less (=0), trade to diploma (=1), advanced diploma/diploma/associate degree (=2) and university (=3).

Household income was divided by an equivalence factor using the Organization for Economic Cooperation and Development (OECD) modified scale (Hagenaars et al. 1994). The equivalence factor is the sum of allocated points to household members (i.e. 1 point for the first adult, 0.5 points to each additional person age 15 years and over and 0.3 to each child under the age of 15 years). Equivalised household income was then grouped into four approximately equal quintiles from lowest to highest (lowest = 0, Lower = 1, Higher = 2, Highest = 3).

Dental insurance status was dichotomised as No (=0) or Yes (=1). The variable was derived from three NSAOH questions: ‘Do you have a private health insurance other than Medicare?’ if the response was ‘yes’ or ‘don't know’, participants were asked, ‘What type of private medical insurance do you have?’ if people reported having extras or responded ‘don't know’, they were asked, ‘Does your private health insurance providers cover dental services?’ People who responded ‘yes’ to the last question were classified as having private dental insurance otherwise, as ‘no’.

Concession card ownership indicates eligibility for government assistance and the entitlement to receive public dental services. This is also a proxy measure of socioeconomic status. The variable results from the question, ‘Do you currently receive a pension or allowance from the Government, or have a pensioner concession card, a Health Care Card or a Department of Veterans Affairs card?’. It is coded as No (=0) and Yes (=1).

Difficulty paying a $200 dental bill, was taken from the question ‘At most times of the year, how much difficulty would you have paying a $200 dental bill out of your own pocket?’ the response categories were none (=0), some (=1) and a lot (=2).

The Last Dental Visit captured the recent contact and indicates entering the health care system. It stemmed from the question, ‘How long ago did you last see a dental professional about your teeth, dentures or gums?’ and dichotomised options into 12 months and over or never visited (=0) and less than 12 months (=1).

### 3.3 Statistical analysis

Descriptive analyses were conducted to examine the distributions of the outcome, exposure, and covariates, specifically focusing on determining the prevalence of delayed or avoided dental visits due to cost among older Australians in different residential locations, which aligns with the study's first objective. Additionally, cross‐tabulation tables were generated to present the frequencies and percentages of the outcome (avoided or delayed visiting due to cost) by each covariate, addressing the second objective of identifying and comparing sociodemographic and economic factors contributing to financial delays or avoidance of dental services among older Australians based on residential location.

*The non‐linear Blinder‐Oaxaca* decomposition analysis^21^ was used to identify factors that explained most of the regional/area inequalities in financial barriers and showed to what extent financial burden on oral health can be explained by residential location inequalities. The *Blinder‐Oaxaca* decomposition is a counterfactual method used to analyse the potential impact of changing a specific variable (i.e. person's residence) on a particular outcome (i.e. financial difficulty accessing dental services). It breaks down the difference in mean values of the outcome between groups (rural vs. major cities residents) and attributes the changes to the variable in question while holding all other factors constant.^22^, ^23^

Sensitivity analyses were conducted under the four different scenarios using four separate samples: (1) Scenario I: Major city and Rural/remote pooled; (2) Scenario II: Major city only; (3) Scenario III: Rural/remote only; Scenario IV: randomized the ordering of variables by using a large number of simulations (*n* = 1000) across all possible ordering of variables.

All analyses were conducted using the weighted data and *Oxaca & Fairlie* command in Stata 11. Detailed formulas and computational coding are presented as appendices in the online Data S1.

## 4 RESULTS

A total of 4103 Australian older adults aged 65 years or over (Mean ± SD: 74.1 ± 6.7), with 53% being female, were interviewed in 2017–18. Over two‐thirds (66%) of the population resided in major cities. Approximately one‐quarter of both major cities and rural residents reported avoiding or delaying dental visits due to cost (25% and 27%, respectively) (Table 1).

**TABLE 1:** Distribution and prevalence of variables among the sample population of Australian adults aged 65+ years (weighted).

	Count = 4103	Proportion %	95% Lower CL for *N* %	95% Upper CL for *N* %
Sex				
Male	1917	46.7	45.2	48.3
Female	2186	53.3	51.7	54.8
Residential location (exposure)				
Regional/remote	1361	33.2	31.7	34.6
Major city	2742	66.8	65.4	68.3
Education level				
Secondary school or less	1428	34.9	33.5	36.4
Trade to diploma	246	6.0	5.3	6.8
Advanced diploma/diploma/associate degree	1931	47.3	45.7	48.8
University	481	11.8	10.8	12.8
Equivalised household income				
Lowest	1499	46.9	45.2	48.7
Lower	1165	36.5	34.8	38.2
Higher	353	11.1	10.0	12.2
Highest	177	5.5	4.8	6.4
Dental insurance				
No	2216	54.5	53.0	56.0
Yes	1848	45.5	43.9	47.0
Government health concession card ownership				
No	754	18.4	17.2	19.6
Yes	3346	81.6	80.4	82.8
Difficulty paying $200 dental bill				
None	1526	37.7	36.2	39.2
Some	1694	41.8	40.3	43.3
Large	832	20.5	19.3	21.8
Last dental visit				
12+ months ago	1688	41.5	39.9	43.0
<12 months ago	2384	58.5	57.0	60.1
Avoided or delay dental visit due to cost (outcome)				
No	3007	73.5	72.2	74.9
Yes	1082	26.5	25.1	27.8

### 4.1 Prevalence of avoided or delayed dental visits

The prevalence of avoiding or delaying dental visits due to cost varied significantly by demographic and socioeconomic factors (Table 2). Females (56%) were more likely to report this issue than males (45%). Financial barriers were particularly pronounced among those with lower income and no dental insurance. Specifically, 59% of individuals with the lowest income and 33% with lower income reported avoiding or delaying visits, compared to only 2% and 5% in the highest and higher income brackets. Similarly, 77% of those without dental insurance avoided or delayed visits, compared to 23% with insurance.

**TABLE 2:** Distribution of individuals who reported avoided or delayed dental visits or treatments among Australian adults aged 65+ years (weighted).

	Avoided or delayed visiting due to cost *n* (%)	
	No	Yes
Sex		
Male	1431 (47.6)	481 (44.5)
Female	1576 (52.4)	601 (55.5)
Residential location (exposure)		
Regional/remote	976 (32.5)	377 (34.8)
Major city	2031 (67.5)	705 (65.2)
Education level		
Secondary school or less	999 (33.3)	425 (39.5)
Trade to diploma	175 (5.8)	69 (6.5)
Advanced diploma/diploma/associate degree	1448 (48.3)	477 (44.4)
University	376 (12.5)	103 (9.6)
Equivalised household income		
Lowest	966 (42.1)	528 (59.3)
Lower	862 (37.5)	300 (33.7)
Higher	308 (13.4)	44 (5.0)
Highest	160 (6.9)	18 (2.0)
Dental insurance		
No	1386 (46.6)	824 (76.6)
Yes	1590 (53.4)	252 (23.4)
Concession health card ownership		
No	616 (20.5)	137 (12.7)
Yes	2388 (79.5)	944 (87.3)
Difficulty paying $200 dental bill		
None	1377 (46.3)	144 (13.5)
Some	1234 (41.5)	456 (42.7)
Large	361 (12.1)	468 (43.8)
Last dental visit		
12+ months ago	1909 (64.0)	470 (43.8)
<12 months ago	1075 (36.0)	604 (56.2)

Dental insurance coverage was significantly higher in the high‐income (i.e. higher and highest) group (71%) compared to the low‐income (lower and lowest) group (39%). Conversely, concession card ownership was more prevalent among the low‐income group (91%) than the high‐income group (41%).Cross‐tabulation results between income, insurance and concession card ownership are available in Data S1.

### 4.2 Socioeconomic factors and regional differences

*The non‐linear Blinder‐Oaxaca* decompositionanalysis results (Table 3) indicated that 30% of the regional/remote population and 26% of the major city population reported avoiding or delaying dental visits due to cost.

**TABLE 3:** Non‐linear Blinder‐Oaxaca decomposition of the change in the prevalence of avoided or delayed dental visiting due to cost among Australian adults aged 65+ year old in 2017–18 (weighted).

	Estimate (95% CI)	
Prevalence (%) of avoided or delayed dental visiting due to cost (rural/remote)	30.1 (26.9, 33.3)	
Prevalence (%) of avoided or delayed dental visiting due to cost (major city)	26.2 (23.7, 28.7)	
Row difference	0.0391 (0.0002, 0.0798)	
Due to endowments (E)	0.0205 (0.0011, 0.0402)	
Due to coefficients (C)	0.0201 (−0.0187, 0.0590)	
Due to interaction (CE)	−0.0011 (−0.0132, 0.0101)	
% Explained	53.8	
% Unexplained	46.2	
Explanatory variables		
	Estimate due to difference in characteristics	
	Estimate (95% CI)	Proportion explained (%)
Sex	−0.0002 (−0.0026, 0.0021)	0.0
Education level	−0.0062 (−0.0131, 0.0003)	−30.6
Equivalised household income	0.0030 (0.0000–0.0058)	14.9*
Government health concession card ownership	−0.0019 (−0.0070, 0.0031)	−9.4
Dental insurance	0.0085 (0.0038–0.0130)	42.1**
Last dental visit	0.0042 (0.0000–0.0084)	20.8*
Difficulty paying $200 dental bill	0.0126 (0.0091–0.0163)	62.4**

* *p*‐value <.05.

** *p*‐value <.001.

The analysis attributed the differences in prevalence to three factors: Endowments (E), Coefficients (C), and their interaction (CE).^24^ Endowments (unchanging characteristics) and coefficients (how characteristics influence outcomes) contributed 0.0205 and 0.0201, respectively, while the interaction term contributed −0.0011. Overall, 53.8% of the disparities in prevalence were explained by these variables, leaving 46.2% unexplained.

The largest explanatory variable was difficulty paying a $200 dental bill, which accounted for 62.4% of the differences. Dental insurance, last dental visit and equivalised household income also played a significant role, explaining 42.1%, 20.8% and 14.9% of the differences, respectively. The remaining variables, such as government health concession, gender and education explained a relatively small in significant proportion of the differences.

### 4.3 Sensitivity analysis

The sensitivity analysis (Data S1) confirmed the robustness of the findings. The prevalence difference between rural/remote and major city areas was 4%, consistent with the original estimates. The explained disparities ranged from 46% to 54%.

- Scenario I and II: Estimates and variable distributions were similar to the original.
- Scenario III: Showed a marginal reduction in prevalence difference, contributing to a decrease in the explained percentage from 53.8% to 46.2%.

Examining the explanatory variables, sex exhibited a negligible effect, education level showed a more pronounced impact with a decrease of 10.3% in Scenario I and 28.9% in Scenario II, while equivalised household income revealed a positive association, increasing by 10.9% in Scenario I and 12.9% in Scenario II. Government health concession card ownership displayed a negative association, contributing to a prevalence decrease of 23.9% in Scenario I and 8.4% in Scenario II.

Dental insurance had a substantial positive impact, with a prevalence increase of 41.3% in Scenario I and 40.4% in Scenario II. Last dental visit and difficulty paying a $200 dental bill both demonstrated positive associations, contributing to an increase in prevalence.

## 5 DISCUSSION

This study implemented descriptive and non‐linear Blinder and Oaxaca decomposition analyses to investigate the factors contributing to the disparities in the prevalence of avoided or delayed dental visits due to cost between regional/remote and major city older adult populations in Australia.

Findings show that financial barriers in utilizing dental services for older Australians are prevalent, as almost one in three reported avoiding or delaying dental visits due to cost.

Several associated factors contribute to the disparities in accessing dental care among older adults in Australia. Evidence explains some potential reasons for explaining this financial burden in terms of utilizing dental services for older Australians residing in both major cities and rural areas. According to the Australian Institute of Health and Welfare (AIHW), older adults are more likely than younger individuals to live in low‐income households. Specifically, 66% of households with a reference person aged 65 years or older fall into the lowest 40% of households ranked by equivalised disposable income.^25^ Combining this with the fact that the tax rebate for private insurance is more in favour of higher‐income individuals,^26^ a higher financial burden associated with utilizing dental services for older Australians is explicable.

According to the descriptive results, older adults with the lowest and lower equivalised household income were more likely to avoid or delay dental visits than those with higher income levels. Similarly, those without dental insurance were more likely to avoid or delay dental visits compared to those with dental insurance. These findings, consistent with the findings from the decomposition analysis and with previous research, suggest that improving access to dental insurance and addressing financial barriers to dental care could reduce disparities in accessing dental care among older adults.^27^, ^28^

The results of the decomposition analysis offer valuable insights into how various established factors contribute to the prevalence of avoiding or delaying dental visits among older adults in Australia. Specifically, the analysis showed that differences in ability to pay a $200 dental bill, dental insurance, last dental visit and equivalised household income explained a large proportion of the disparities, indicating that addressing these factors could be important in reducing disparities in accessing dental care.

Decomposition analysis did not support that ownership of a government health concession card statistically explained disparities in avoided or delayed dental visits due to cost. This may be attributed to the substantial proportion of the high‐income group owning a concession card, highlighting the need to reconsider the concession card mechanism in Australia, as supported by existing literature.^17^

Avoided or delayed dental visits due to cost is more prevalent in older adults in rural areas, indicating that older adults living in rural areas face more barriers to accessing dental care than those living in major cities.

According to the decomposition analysis, the significance of the last dental visit variable was high for both major cities and rural residents, indicating that entering the oral health system plays an important role in explaining disparities in avoiding or delaying dental visits due to cost. Previous Australian research showed that older adults living in rural areas were less likely to have regular dental visits.^29^ Considering the reported association between regular dental visits and dental health conditions,^30^, ^31^ promoting regular dental visits may be an effective strategy for reducing disparities in accessing dental care among older rural residents.

It is worth noting that many public dental services are concentrated in major cities.^14^ According to the findings of the current study, older adults in rural areas reported more prevalent avoidance or delays in dental visits due to cost. This indicates that older adults living in rural areas face more barriers to accessing dental care than those living in major cities. This highlights the significance of expanding the coverage of public dental services to decrease the disparities in dental service utilization based on residential location. Additionally, further investigation is needed to assess the geographical accessibility of public dental services, which would further inform strategies to reduce these disparities.

According to the findings, a significant portion of the disparities remained unexplained, suggesting that factors beyond those associated solely with residential location inequalities may also contribute. Geographical isolation, limited healthcare infrastructure, access to dental and general practitioner services, variations in models of care and dental fee schedules and the availability of social and community support resources likely play crucial roles in shaping these discrepancies.^10^, ^11^ For instance, over 1 in 4 (27%) older Australians received no assistance, with the proportion varying by remoteness: 1 in 3 (35%) in Outer regional, remote and very remote areas, 2 in 7 (28%) in Inner regional areas and 1 in 4 (25%) in Major cities did not receive social and community support.^25^ These unexplained variations underscore the need for further research to comprehensively understand and address these disparities. Future studies should focus on investigating the combined impact of these factors and their interactions to design targeted interventions.

The study has several strengths that enhance the quality of the findings. The use of a nationally representative sample increases the generalizability of the results to the Australian population. Also, following the Blinder‐Oaxaca counterfactual decomposition analysis is a robust method for identifying factors that explain inequalities and the potential impact of changing specific variables.

However, this study also has some limitations that need to be acknowledged. Firstly, the data used are cross‐sectional, meaning causal relationships cannot be inferred due to the inability to establish temporal sequence or account for dynamic changes over time. Secondly, the study only examined financial barriers to accessing dental care and did not consider other potential barriers, such as transportation, mobility and oral health literacy. Furthermore, In Blinder‐Oaxaca decomposition analysis, variables that may serve both as exposure and mediator can distort estimates, complicating the assessment of each factor's independent contribution. Future research using structural equation modelling or longitudinal analysis could clarify causal pathways over time. Causal mediation analysis could also deepen insights into outcome mechanisms. Finally, the study relied on self‐reported data, which may be subject to social desirability bias or measurement error.

In conclusion, disparities in the prevalence of avoided or delayed dental visits due to cost between regional/remote and major city populations are partially explained by a combination of population characteristics and model parameters, but other unexplained factors associated with residential location inequalities may also be involved.

The study suggests that expanding public dental service coverage and reviewing the concession card mechanism in Australia could decrease disparities in dental service utilization based on residential location. Promoting regular dental visits may also be effective in reducing disparities in accessing dental care in rural residents.

## FUNDING INFORMATION

Data gathered from the National Study of Adult Oral Health (NSAOH) were analysed in this study. NSAOH was supported by National Health and Medical Research Council (NHMRC) (Partnership Grant #1115649). The contents are solely the responsibility of the administering institution and authors and do not reflect the views of NHMRC.

## CONFLICT OF INTEREST STATEMENT

LJ is one of the associate editors of the Community Dentistry and Oral Epidemiology Journal.

## Supporting information


Data S1.

## Data Availability

Research data are not shared, but the codes used and responses to the reviewers are publicly available in the supplement file.
