# Supplementary material for: Remoteness‐attributable financial inequality in dental service utilization in Australian older adults: A Blinder‐Oaxaca decomposition
Source: Community Dent Oral Epidemiol. 2024 Aug 18;53(1):42–9. doi: 10.1111/cdoe.13004 (PMC11754145; doi:10.1111/cdoe.13004)
Supplement: Supplementary file 1 — Data S1. [file CDOE-53-42-s001.docx]

**Remoteness-attributable financial inequality in dental service utilization in Australian older adults: A Blinder-Oaxaca decomposition**

Arash Ghanbarzadegan^1,2,3^, Xiangqun Ju ^1^, Woosung Sohn^2^, Lisa Jamieson^1^

1. Australian Research Centre for Population Oral Health (ARCPOH), Adelaide Dental School, The University of Adelaide, Adelaide, Australia
2. Population Oral Health, Sydney Dental School, Faculty of Medicine and Health, The University of Sydney, Australia
3. Menzies Centre for Health Policy and Economics, School of Public Health, The University of Sydney, Australia

*Corresponding Author's contact details

**Email:** [arash.ghanbarzadegan@adelaide.edu.au](mailto:arash.ghanbarzadegan@adelaide.edu.au)

**Phone:** +61883132895

**Address:** ARCPOH, Level 4, Rundle Mall Plaza, The University of Adelaide, Rundle Mall, Adelaide 5000 SA, Australia

**Appendix Table 1- Cross-tabulation between Equivalised household income and insurance among the sample population of Australian adults aged 65+ years (weighted valid cases)**

|  | | Dental Insurance Status | | | |
| --- | --- | --- | --- | --- | --- |
|  |  | No | | Yes | |
|  |  | Count | Row N % | Count | Row N % |
| Equivalised household income | Lowest | 963 | 64.8% | 524 | 35.2% |
|  | Lower | 639 | 55.3% | 516 | 44.7% |
|  | Higher | 105 | 29.8% | 247 | 70.2% |
|  | Highest | 44 | 24.9% | 133 | 75.1% |

**Appendix Table 2- Cross-tabulation between Equivalised household income and concession card ownership among the sample population of Australian adults aged 65+ years (weighted valid cases)**

|  | | Government health concession card ownership | | | |
| --- | --- | --- | --- | --- | --- |
|  |  | Yes | | No | |
|  |  | Column N % | Count | Column N % | Count |
| Equivalised household income | Lowest | 52.5% | 1396 | 19.2% | 102 |
|  | Lower | 39.3% | 1045 | 22.4% | 120 |
|  | Higher | 6.9% | 183 | 31.8% | 170 |
|  | Highest | 1.3% | 35 | 26.6% | 142 |

**Appendix Table 3: Non-linear Blinder-Oaxaca decomposition of Rural/remote and Major city gap in the prevalence of avoided or delayed dental visiting due to cost under different scenarios among Australian adults aged 65+ year old in 2017-18 (weighted)**

|  | Scenario I | | Scenario II | | Scenario III | | Scenario IV | |
| --- | --- | --- | --- | --- | --- | --- | --- | --- |
|  | Estimate (95% CI) | % | Estimate (95% CI) | % | Estimate (95% CI) | % | Estimate (95% CI) | % |
| Prevalence (%) of avoided or delayed dental visiting due to cost (Rural/remote) | 30.1 (26.9, 33.3) |  | 30.1 (26.9, 33.3) |  | 30.1 (26.9, 33.3) |  | 30.1 (26.9, 33.3) |  |
| Prevalence (%) of avoided or delayed dental visiting due to cost (Major city) | 26.2 (23.7, 28.7) |  | 26.2 (23.7, 28.7) |  | 26.2 (23.7, 28.7) |  | 26.2 (23.7, 28.7) |  |
| Row difference | 0.0391 (0.0002, 0.0798) |  | 0.0391 (0.0002, 0.0798) |  | -0.0391 (-0.0802, 0.0020) |  | 0.0391 (0.0002, 0.0798) |  |
| % Explained | 0.0193 (0.0018, 0.0378) | 48.7 | 0.0205 (0.0008, 0.0400) | 53.8 | -0.0179 (-0.0421, -0.0008) | 46.2 | 0.0184 (0.0011, 0.0376) | 46.2 |
| **Explanatory variables** | | | | | | | | |
| Sex | -0.0011 (-0.0029, 0.0008) | -6.0 | -0.0002 (-0.0027, 0.0022) | -0.8 | 0.0005 (-0.0006, 0.0016) | -2.7 | -0.0012 (-0.0031, 0.0007) | -6.7 |
| Education level | -0.0019 (-0.0048, 0.0009) | -10.3 | -0.0063 (-0.0130, 0.0004) | -28.9 | 0.0006 (-0.0022, 0.0034) | -3.3 | -0.0007 (-0.0038, 0.0025) | -3.9 |
| Equivalised household income | 0.0020 (0.0005, 0.0034) | ^**^10.9 | 0.0029 (0.0000, 0.0058) | ^*^12.9 | -0.0060 (-0.0116, -0.0004) | ^*^33.0 | 0.0039 (0.0000, 0.0078) | ^*^21.7 |
| Government health concession card ownership | -0.0044 (-0.0083, -0.0004) | ^*^-23.9 | -0.0019 (-0.0071, 0.0032) | -8.4 | 0.0060 (-0.0004, 0.0124) | -33.0 | -0.0060 (-0.0121, 0.0001) | -33.3 |
| Dental Insurance | 0.0076 (0.0049, 0.0103) | ^***^41.3 | 0.0091 (0.0044, 0.0139) | ^***^40.4 | -0.0175 (-0.0242, -0.0109) | ^***^96.2 | 0.0124 (0.0068, 0.0179) | ^***^68.9 |
| Last dental visit | 0.0035 (0.0012, 0.0057) | ^**^19.0 | 0.0046 (0.0000, 0.0092) | ^*^20.4 | -0.0086 (-0.0145, -0.0028) | ^**^47.3 | 0.0064 (0.0015, 0.0112) | ^*^35.6 |
| Difficulty paying $200 dental bill | 0.0127 (0.0103, 0.0151) | ^***^69.0 | 0.0143 (0.0156, 0.0181) | ^***^63.6 | 0.0068 (0.0042, 0.0095) | ^***^-37.4 | 0.0032 (-0.0001, 0.0065) | 17.8 |
| Total |  | 100 |  | 100 |  | 100 |  | 100 |

Notes: Scenario I: Major city and Rural/remote pooled; Scenario II: Major city only; Scenario III: Rural/remote only; Scenario IV: randomized the ordering of variables by using a large number of simulations (n=1000) across all possible ordering of variables.

***p-value<0.001; **p-value<0.01; *p-value<0.05.

**Stata Code:
Variables:**

Y= Avoided or delayed visiting due to cost

X=Residential location

C_1_=Sex

C_2_= Equivalised household income

C3=Dental insurance

C_4_= Government health concession card holder

C_5_= Difficulty paying $200 dental bill

C_6_= Last dental visit

PW=Weight

**Stata codes:**

decompose Y C_1_ C_2_ C_3_ C_4_ C_5_ C_6_ [pw= weight], by (X) detail estimates

Oaxaca Y C_1_ C_2_ C_3_ C_4_ C_5_ C_6_ [pw= weight], by( X) logit
